# Intergenerational Exchanges In Aging South Asian Muslim Families: An Intersectional Lifecourse Perspective

**DOI:** 10.1093/geroni/igab046.1244

**Published:** 2021-12-17

**Authors:** Mushira Khan, Karen Kobayashi, Andre Smith

**Affiliations:** 1 University of Victoria, Plainfield, Illinois, United States; 2 University of Victoria, Victoria, British Columbia, Canada

## Abstract

International migration flows are increasing at a rapid pace and are often accompanied by emergent global realities, (re)negotiation of identities and familial bonds, anticipated challenges, and unforeseen exigencies. Concomitantly, advances in public health have resulted in longer lives with an increasing proportion of the global population now 65 years and older. While these demographic shifts have received considerable research attention, little is known about aging South Asian Muslim families in the US and the ways in which they adjust and adapt to shifting social realities. To address this gap, this qualitative study explores the intersections of faith, culture, gender, age, and immigrant status, and how these seminal life course events shape intergenerational care and support exchanges in South Asian Muslim families. Building on findings from 30 in-depth narrative interviews with three generations of immigrant South Asian Muslim women, and using an intersectional lifecourse perspective, this study explores the (re)negotiation of familial bonds and the enactment of religious beliefs and practices such as those around filial expectations in a transnational Islamic context. It shows how, for the grandmothers, daughters, and granddaughters in the study, their Islamic faith was a part of both the public sphere and a collective ideology, as well as a deeply personal and intimate attachment that provided structure and continuity in their everyday lives. Finally, the implications of these findings in the broader context of Islamophobia and salient structural barriers to accessing available health and social support services for aging South Asian Muslim families are discussed.

